# Biologically based neural circuit modelling for the study of fear learning and extinction

**DOI:** 10.1038/npjscilearn.2016.15

**Published:** 2016-11-09

**Authors:** Satish S Nair, Denis Paré, Aleksandra Vicentic

**Affiliations:** 1 Department of Electrical and Computer Engineering, University of Missouri, Columbia, MO, USA; 2 Center for Molecular and Behavioral Neuroscience, Rutgers University—Newark, Newark, NJ, USA; 3 Division of Neuroscience and Basic Behavioral Science, National Institute of Mental Health, Rockville, MD, USA

## Abstract

The neuronal systems that promote protective defensive behaviours have been studied extensively using Pavlovian conditioning. In this paradigm, an initially neutral-conditioned stimulus is paired with an aversive unconditioned stimulus leading the subjects to display behavioural signs of fear. Decades of research into the neural bases of this simple behavioural paradigm uncovered that the amygdala, a complex structure comprised of several interconnected nuclei, is an essential part of the neural circuits required for the acquisition, consolidation and expression of fear memory. However, emerging evidence from the confluence of electrophysiological, tract tracing, imaging, molecular, optogenetic and chemogenetic methodologies, reveals that fear learning is mediated by multiple connections between several amygdala nuclei and their distributed targets, dynamical changes in plasticity in local circuit elements as well as neuromodulatory mechanisms that promote synaptic plasticity. To uncover these complex relations and analyse multi-modal data sets acquired from these studies, we argue that biologically realistic computational modelling, in conjunction with experiments, offers an opportunity to advance our understanding of the neural circuit mechanisms of fear learning and to address how their dysfunction may lead to maladaptive fear responses in mental disorders.

## Introduction

Recently, neuropsychiatry has undergone a major shift in perspective from diagnostic entities rooted in checklists of symptoms to one centred on measurable behavioural and cognitive dimensions, which different psychiatric categories might share. The implementation of this new strategy, termed research domain criteria (RDoC) project, involves a new classification system for clinical research on mental disorders that is explicitly dimensional in its approach. Currently, RDoC includes five dimensions of functioning, one of which is the negative valence system (http://www.nimh.nih.gov/research-priorities/rdoc/negative-valence-systems-workshop-proceedings.shtml). This system is thought to be responsible for responses to aversive events and situations, including responses to acute threat (fear), responses to potential harm (anxiety), responses to sustained threat, frustrative non-reward and loss. Although neuroscientists have been studying this system intensely for decades, we still have a limited understanding of how distributed neuronal activity in fear/anxiety networks influence behaviour. In part, this situation results from the complexity of the nervous system and the difficulty of integrating vast amounts of research data obtained at different levels of analyses. In this commentary, we argue that computational modelling grounded in biological information constitutes a promising path towards a more integrated understanding of fear/anxiety networks.

## The challenge

Unquestionably, neuroscientists have made immense progress in characterising the neural substrates of the negative valence system and data continues to accumulate at an astounding pace. For instance, key nodes in the network have been identified, their major interconnections mapped out, and a crude understanding of their influence on behaviour is emerging. Within each of these nodes, multiple cell types have been identified and their physiological properties as well as pharmacological responsiveness have been characterised to various degrees. In addition, the impact of several genetic variations on negative emotional behaviours has been analysed. Yet, a precise understanding of the mechanisms and computations allowing these networks to flexibly regulate emotional expression still eludes us.

Biologically based neural circuit modelling is a promising, yet so far neglected, approach that could assist us in understanding how the neural circuits that subserve emotion process, represent and store information. Consider the case of Pavlovian fear conditioning (note that we use the term fear as a shorthand for defensive behaviors).^1^ Although it is one of the oldest and most extensively studied tasks of aversive learning, its neurobiological bases are still not fully understood. In this laboratory model, a neutral sensory stimulus (CS) develops the ability to elicit conditioned defensive behaviours (CRs) such as behavioural freezing, after being paired a few times with a noxious unconditioned stimulus (US). When neuroscientists began studying this form of emotional learning, optimism was high that it would be rapidly understood because of its apparent simplicity. And indeed, early models^2^ suggested that Pavlovian fear learning depended on a simple mechanism, located entirely in the amygdala: information about the CS and US would converge in the lateral amygdala (LA), causing the NMDA-dependent reinforcement of CS inputs. As a result, later CS presentations would excite LA neurons more strongly, causing them to elicit CRs through their projections to the central amygdala (CeA).

However, subsequent research revealed that fear learning involves a much more complex circuit than initially conceived. In particular, it was realised that LA is not the only site of plasticity for Pavlovian fear: amygdala-projecting auditory thalamic and cortical neurons also display increases in CS responsiveness that are critical for fear learning.^3,4^ Even CeA, initially envisioned as a passive output station to downstream fear effectors, emerged as yet another critical site of plasticity.^5–7^ Within the amygdala, it was also discovered that several parallel inhibitory and excitatory circuits are differentially involved^8^ and regulated by medial prefrontal neurons during the expression or extinction of conditioned fear.^9^ More recently, it was realised that the fear memory trace is not fixed but that it displays a changing dependence on different brain structures as a function of time since conditioning.^10,11^

As the example of Pavlovian fear illustrates, it will be extremely challenging to understand fear/anxiety networks because they are complex dynamical systems with multiple feedback loops that operate on different time scales and at multiple levels. These include molecular interactions within single cells, a complex interplay between multiple cell types within and between each network node, the dynamical properties of synaptic transmission at each site and for each synapse type, as well as multiple interacting neuromodulatory mechanisms that act pre- and post-synaptically, all ultimately working together to determine behaviour in a way that defies intuitive understanding.

Here we reason that uncovering these complex neurobiological relations is insurmountable without powerful computational modelling tools and the conceptual frameworks they provide. Indeed, biologically based modelling can be used to integrate biological information at different levels of organisation. As a result, they allow the study of emergent properties that intuition cannot readily predict. Importantly, these models are not fixed but can be updated as new experimental data become available. Ideally, a reciprocal interaction should exist between models and experiments such that experimental observations serve to both constrain the model and verify its predictions. At the same time, they provide a rapid means to quantitatively test various hypotheses that would be difficult to address experimentally.

Notable examples at the single cell and small circuit levels include synaptic integration,^12,13^ cerebellar computations^14^ and the genesis of neuronal oscillations in various networks.^15,16^ At the level of higher cognitive processes, biophysically realistic models of cortical networks with recurrent connectivity and leaky integrate-and-fire neurons were able to reproduce the activity of cortical cells during decision-making. They also suggested a cellular mechanism for the accumulation of evidence over time, whereby recurrent excitation in concert with NMDA receptor activation could create attractor states that support decision-making, and potentially, working memory.^17^

In contrast, there is a dearth of computational modelling studies devoted to investigating the functions of the amygdala and its target networks in fear learning. Although manipulations of specific cell types and neural projections are moving the field forward, experimental studies are not suited to identify circuit-level mechanisms that can predict and explain behaviour. Computational approaches are ideally positioned to probe these mechanisms and quantitatively determine critical properties of amygdala circuitry across levels of investigation, while helping to form more specific theories regarding the link between circuit dysfunctions and mental disorders. Convergent computational–experimental approaches will also be critical in studying the impact of negative valence stimuli on other forms of learning and decision-making.

## Different approaches to modelling

Nervous system function can be studied at several levels, from the molecular, cellular and network levels to the cognitive and behavioural levels. For instance, single-nucleotide polymorphisms in various genes correlate with trauma susceptibility and abnormalities in fear acquisition or extinction.^18^ However, finding the mechanistic links between these gene polymorphisms and alterations in circuit behaviour or cognition represents a formidable challenge, even from a modelling perspective. Therefore, this paper focuses on modelling at the synaptic, cellular and systems level. Readers are referred to recent reviews addressing modelling at the molecular level^19^ or how cognitive models can help guide lower level models.^20^

### Neuronal level

Historically, computational models have represented neurons in various ways. For instance, integrate-and-fire neuron models were introduced more than a century ago,^21,22^ but limitations in our understanding of neurophysiology slowed their acceptance until the 1980s. In contrast, connectionist and parallel distributed processing models^23^ (now categorised under *data-driven* machine learning methods^24^) rapidly gained popularity because of their ability to capture psychological phenomena. On the basis of abstract representations of neuronal properties and connectivity patterns,^25^ these models were successful in mapping complex input–output relationships using minimal networks of neuronal units. Importantly, they provided a framework to study salient aspects of information processing such as stability, convergence time, storage capacity and size-based scaling of properties.

However, the rapid growth in neurophysiological data and computational power over the past few decades has renewed interest in theory-driven realistic computational models. Two popular examples of this type include models where neurons are represented by firing rate and integrate-and-fire formulations.^26^ Izhikevich neurons represent another formulation where the transition from sub- to supra-threshold activity as well as other dynamical properties like bursting and spike frequency adaptation are modelled using mathematical insights derived from bifurcation theory.^27^ Such models have been used to explore theoretical issues related to network structure, stability and oscillatory potentials.

Although the above models can reproduce various aspects of neuronal behaviour, they do so using abstract representations, not by directly integrating the neurons’ biophysical properties. Indeed, different types of neurons display particular sets of ionic conductances whose kinetics and distribution shape their spontaneous activity and constrain their synaptic responsiveness. These distinct electroresponsive properties translate into characteristic activity patterns such as subthreshold oscillations, post-inhibitory firing, as well as bistability of resting and spiking states. Biologically based formulations that incorporate these biophysical properties are increasingly being used in modelling studies and these can provide cross-level understanding of brain dynamics and behaviour.

In these models, ionic channels are represented using the Hodgkin–Huxley formulation.^28^ Depending on the application, these models can range tremendously in their level of detail. At one pole are models where neurons are represented as a single compartment with just a few of the main types of ionic channels they are known to express. At the other are models that feature the full morphological and biophysical complexity of neurons. For instance, a rich repertoire of neuronal reconstructions (including 3D rendering of soma, dendrites and sometimes axons) for both principal excitatory (stellate and pyramidal) cells and GABAergic inhibitory interneurons of the rodent amygdala are available free to modelers from neuromorpho.org. Generally, the level of detail incorporated in such models is a function of the questions investigated and associated computational demands. For instance, models investigating the integrative properties of single cells use detailed multi-compartmental models that feature an exhaustive representation of active dendritic properties^29,30^ and their complex role in information processing.^13^ In contrast, simpler models featuring one or just a few morphological compartments are favoured for studies of complex networks where simulating interactions between large populations of neurons are computationally intensive.^31^

### Synaptic level

Using the same type of formulation, these models can also integrate detailed biophysical information at the synaptic level, including reversal potential, rise and decay times of synaptic conductances, short-term dynamics of transmitter release probability (facilitation and depression), and activity-dependent long-term plasticity. As for all other aspects, these factors are adjusted to match experimental findings obtained in the specific cell type to be modelled or, if unavailable, a close relative. Although these variables have considerable influence on network activity, including in fear and extinction learning,^32^ the specifics of how they affect such activity are only beginning to be understood.

### Network level

At the network level, models typically follow the ‘network paradigm’, which attributes the information processing capability of neuronal systems not to the properties of individual neurons, but to their intricate connections. Ideally, models should ‘incorporate the vast richness of the structural and physiological properties of real neurons and synapses’,^33^ and this is facilitated by biophysical computational approaches. The generic approach to incorporating such biological realism into network models starts by considering low-level features such as the biophysical properties of each individual cell types, intrinsic and extrinsic connections, and properties of short- and long-term activity-dependent plasticity. While it is usually impossible for models to incorporate as many of the different cell types as exist in the real network, one populates the model with the same proportion of the different cell types and compensates for the reduction in network size by increasing synaptic strengths.^34^ Another important consideration is to reproduce, in computer topology, the spatial distribution of the different cell types. This is critical as axonal conduction times have a critical role in shaping distributed activity patterns in time.

### Algorithmic models and data-driven machine learning approaches

We briefly discuss an alternative theory-driven ‘algorithmic’ approach of the top-down and intermediate level (between biological and pure behavioural levels) type, termed reinforcement learning, that has been proposed to bridge the gap between specific neurophysiological correlates and pathology in psychiatry.^24,35^ The technique has been applied to model reward-motivated behaviour using abstract mathematical equations and used in normative decision-making frameworks of mental dysfunction.^36^ These authors advocate new types of phenotyping approaches to estimate parameters in models of human decision-making, taking advantage of the fact that aberrant decision-making is central to the majority of psychiatric conditions. A related approach, using Bayesian belief theory, suggests that the brain engages in active inference to formalise perception and behaviour.^37^ In this scheme, higher cortical levels generate top-down predictions of representations at lower levels, which are then compared with actual lower level representations to form a prediction error. In turn, this error is used to update representations up the hierarchy, and the recursive process continues until representations stabilise at all levels. Such Bayesian belief frameworks have identified disruptions in decision-making caused by schizophrenia and autism. Interestingly, such algorithmic models and data-driven machine learning approaches are increasingly using biological information (e.g., brain anatomy and receptors), while, on the other hand, biologically based models are being reduced to ‘mean-field’ population rate models similar to connectionist types.^35^ A recent direction, enabled by big data, aims to develop model-based diagnosis in psychiatry using extremely large data sets, using both data-driven machine learning and the theory-driven biologically based and algorithmic approaches discussed in this paper.^24,35^ For example, models that employ the 'deep learning' algorithm are based on neural networks with the goal of revealing higher level attributes from data.^38^ Another promising branch of machine learning is statistical learning, which encompasses methods that investigate and integrate structure from data that are replicable across different samples obtained from the same population. These can enhance our understanding of big data sets obtained from both basic and clinical studies.^39^ Although not yet used to study fear and extinction learning, these alternative approaches have potential to provide top-down perspectives, and to supplement biologically based models in helping to unravel the role of maladaptive fear responses in mental disorders.

### Constraining the model when experimental data are lacking

Although single-cell models can be developed with reasonable fidelity using experimental data, network parameters such as connectivity, synaptic efficacies, learning mechanisms and neuromodulation are often not characterised as fully. When experimental data are lacking, these then need to be adjusted to match the behaviour of the real network. In such cases, for each low-level property, the experimental literature is searched for constraints and model parameters are varied within reasonable bounds until the network behaviour replicates prior experimental findings, independent of other low-level model properties (see Box 1 for a brief overview of the modelling process). Because there are typically no quantitative data about the spatial distributions of inputs and outputs, connections in such models are typically set with probabilistic gradients of connectivity. Note that in large model networks, such as the LA network developed by Kim *et al.* ^40^ there are so many neurons (~1,000) and synapses (~40,000) that individual low-level aspects cannot be tuned to achieve particular impacts on high-level model behaviour.

### Assessing the model’s validity

While building a stable model that satisfies most available constraints is challenging, it is even more difficult to assess whether its ‘output’ makes sense. It is difficult to understand a network without knowing what it is computing. This problem is particularly acute when the modelled networks regulate responses that do not easily lend themselves to quantification such as those generated by the negative valence system. So far, modelling studies in this field have used the magnitude of neuronal responses to the CS as a proxy for the intensity of emotional responses.^40–42^ In addition, to assess the network’s output specificity, some have contrasted network responses to a range of stimuli that display a gradient of similarity to the original CS.^43^ Although these output measures are adequate for simulating the impoverished circumstances of classical fear conditioning, they will not suffice when studying more complex and realistic behavioural paradigms.

## Prior computational models of pavlovian fear and extinction

Computational models related to fear and extinction circuits are only beginning to emerge. Indeed, a literature search in Pubmed using the search terms ‘computational’, ‘model’ and ‘amygdala’ yielded ~30 times fewer citations (57) than with ‘cortex’ (1,744). Moreover, computational models of other networks have investigated fundamental mechanisms that likely have direct relevance to the negative valence system, including pattern recognition/completion, genesis and synchronisation of neuronal oscillations, as well as experience-driven synaptic plasticity. In this section, we provide a brief review of representative fear models, emphasising biophysical models.

### Connectionist models

Paralleling the development of computational models in other brain areas, one of the first models of Pavlovian fear was an anatomically constrained connectionist model of the network formed by the amygdala, auditory thalamus and cortex.^44,45^ This model could be trained to associate a specific tone CS with a footshock US using a Hebbian-type learning rule, and reproduced conditioning-induced frequency-specific changes in the receptive fields of auditory thalamic^46,47^ and amygdala neurons.^48^ However, this early model lacked biological realism in that it represented each structure using pools of simple nonlinear units (output from 0 to 1, representing average firing rate) and did not separate different sub-nuclei within the amygdala.

### Firing rate and integrate-and-fire models

More recently, a neurally plausible framework was proposed to reproduce several empirical observations on fear learning, using a conceptual firing rate model formulation.^49^ In this model, fear learning and extinction resulted from neuromodulation-controlled long-term potentiation at thalamic, cortical and hippocampal synapses onto principal and local-circuit cells of the lateral and basal amygdala nuclei. The model included conditioning, secondary reinforcement, blocking, the immediate shock deficit, extinction, renewal and a range of experimental observations such as the effects of pre- and post-training ablation or inactivation of the hippocampus or particular amygdala nuclei. Furthermore, this model made several predictions for phenomena it was not designed to address, particularly with respect to the contextual dependence of extinction. However, being of a ‘top-down’ type model that lacks biological realism, translating this model’s predictions into precise neurophysiological mechanisms is problematic.

Another firing rate model that was constrained to reproduce salient properties of LA neurons^50^ suggested combinations of tone and shock densities that could reproduce experimental estimates of different types of tone responsive cells observed after fear conditioning in the lateral amygdala.^51^ Vlachos *et al.*^52^ developed a network model of leaky integrate-and-fire neurons that reproduced the differential recruitment of two distinct subpopulations of basal amygdala neurons, reminiscent of the fear and extinction neurons observed experimentally.^53,54^

### Realistic biophysical models

So far, realistic biophysical models have been developed for intercalated^55^ and LA^40^ neurons. We briefly describe the latter. The number of LA neurons^56^ was scaled down 30 to 1 in the model, including 800 principal cells and 200 fast-spiking interneurons, which were distributed randomly in a realistic tri-dimensional representation of the horn-shaped LAd. Principal cells were simulated with three compartments that included multiple voltage-dependent currents to match the passive and active membrane properties observed experimentally. By varying the density of Ca^2+^-dependent K^+^ currents, model principal cells also reproduced the continuum of spike frequency adaptation seen in these neurons.^57,58^ Prior experimental observations about the spatially heterogeneous intrinsic connectivity that exists in different parts of LA^59^ were reproduced using probabilistic gradients of excitatory and inhibitory connectivity. Extrinsic inputs included thalamic tone and shock inputs as well as brainstem neuromodulatory (dopaminergic and noradrenergic) inputs adjusted to reproduce experimental observations.^60^ All the glutamatergic synapses in the model could undergo both short-term and long-term activity-dependent plasticity, except for those delivering shock or background inputs. Ca^2+^ entered post-synaptic pools at excitatory synapses via NMDA receptors (and Ca^2+^-permeable AMPA receptors for interneurons^61^) and voltage-gated calcium channels. In turn, the intracellular Ca^2+^ concentration determined the long-term potentiation or depression of the synapses.

Not only could the model replicate the formation of two distinct types of tone-responsive principal cell populations, as observed experimentally by Repa *et al.* ^51^, it also led to new insights in the mechanisms of fear memory formation. Previously, there had been much debate regarding this question, with some emphasising the role of plasticity at afferent auditory inputs^3^ and others of plasticity within the amygdala.^62^ Unexpectedly, the model revealed that both views were correct. Indeed, while increases in the CS responsiveness of auditory thalamic neurons were found to be essential for fear learning in the model, after training they were no longer needed because the fear memory was maintained by post-synaptic increases in synaptic efficacy within LA.

In addition, this biophysically realistic model of the amygdala revealed that fear memory formation involves competitive synaptic mechanisms. Previously, it had been reported that only a minority of LA neurons increase their responsiveness to the CS after fear conditioning (25%, refs 51,63,64) even though most cells receive the required inputs.^51^ Related to this observation, another study showed that LA cells expressing high levels of CREB are preferentially recruited into the fear memory trace.^63,65,66^ Yet, when CREB was overexpressed or downregulated in LA, the proportion of LA neurons incorporated into the memory trace remained constant, which led to the proposal that recruitment of LA neurons into the fear memory trace involves a competitive process.^63^ However, the mechanisms underlying this competitive process remained unclear. Because CREB decreases spike after hyperpolarisations, the modelling study of Kim *et al.* ^42^ considered the possibility that a higher intrinsic excitability confers a competitive advantage to particular LA neurons. Consistent with this view, they observed that only 1% of model LA neurons with high spike frequency adaptation increased their CS responsiveness, compared with >40% of the more intrinsically excitable neurons, a prediction that was subsequently validated experimentally.^67^

However, if this factor (intrinsic excitability) acted independently, CREB overexpression would result in the assignment of a higher number of LA cells to the memory trace. Yet, this is not what was seen experimentally or in the Kim *et al.* ^42^ model (CREB overexpression was simulated by converting less into more excitable cells). This suggested that additional factors are at play in the competitive process. Comparative analyses of the intrinsic connectivity of CS responsive versus non-responsive cells revealed that a major substrate of this competition is the distribution of excitatory connections between principal cells and the amount of di-synaptic inhibition they generate in other projection cells. The model revealed that these two factors interact to enhance the likelihood that some principal cells will fire more strongly to the CS at the expense of others. Effectively, the model showed that subsets of more excitable projection cells band together by virtue of their excitatory interconnections to suppress plasticity in other projection cells via the recruitment of local-circuit cells.^42,68^ Another prediction from the model was that the level of inhibtion in the system controlled the size of the fear memory trace.^68^

## Moving beyond amygdalo-centric accounts of emotional learning with biophysical modelling

Despite major advances in our understanding of fear and anxiety, many aspects remain unclear. The literature is replete with contradictions and unresolved questions, which would benefit from the use of biophysical modelling. Although different mammalian species are often more attuned to distinct types of sensory stimuli, mammals can associate almost any arbitrary CS with a pleasant or aversive US. What network architecture could support such flexibility? Stated differently, how is a stimulus identity code transformed into a behavioural response code? When the view emerged that LA is the site of CS–US convergence required for the Hebbian potentiation of CS inputs, it became natural to think of CS-triggered LA firing as potentiated sensory responses that automatically drive defensive CRs. Soon, this tendency generalised to appetitive conditioning and to downstream amygdala nuclei, like the basal amygdala (BA) nuclei (BA=basolateral (BL) and basomedial (BM) nuclei), which are thought to control response effector neurons. Indeed, BL and BM contains multiple subsets of neurons with largely segregated projections to various subcortical sites (these include the lateral or ventromedial hypothalamus, dorsolateral striatum, nucleus accumbens, the medial part of CeA and bed nucleus of the stria terminalis (BNST)) that likely drive specific aversive or appetitive CRs. For instance, recent studies provided evidence that distinct subsets of basolateral amygdala neurons that contribute differential projections to nucleus accumbens and the central amygdala might mediate appetitive and aversive conditioned responses, respectively.^69,70^ Thus, in this conceptual framework, the question becomes how would the activation of select LA neurons by an arbitrary CS influence the correct subset of BA neurons?

However, recent observations suggest that this is not the right way to frame the problem. By contrasting the CS-related activity of BL neurons when rats produced the expected CR or not, it was found that BL cells activated by appetitive CSs mainly encode behavioural output, not CS identity.^71^ Indeed, the CS-related firing of BL cells varied strongly with conditioned responding: it was absent when rats omitted the CR, present when they emitted it, and associated with a neutral CS when rats mistakenly emitted the CR in response to the wrong CS. At the very least, the strong dependence of BL activity on behaviour irrespective of CS identity suggests that feedforward connectivity from LA to BL can be overridden or gated by other BL inputs. However, these results are also compatible with the possibility that LA does not drive CRs via its projections to other amygdala nuclei, but through other structures. Similarly to learned emotional responses, a recent study of innate fear^72^ further supports the idea of expanding the ‘amygdala-centric’ view by showing that activation of a subset of hypothalamic neurons that anatomically bypass the amygdala can lead to a generalisable emotional state, suggesting the role of a broader network in the regulation of emotional states. Note that this departure from amygdalo-centric accounts of learned emotional responses is consistent with earlier work showing that neuronal activity at multiple sites, such as the auditory cortex, posterior and midline thalamic nuclei as well as the prefrontal cortex, is required for the formation or expression of conditioned emotional behaviours.

The fact is that fear researchers, present authors included, have been looking for simple cellular mechanisms, such as Hebbian plasticity at a specific set of synapses, to account for fear learning and have neglected incontrovertible evidence that such mechanisms are incompatible with basic properties of fear memories. Case in point: a basic tenet of memory research in general and of Pavlovian fear learning in particular is Hebb’s idea that coincident activity favours synaptic strengthening. Yet, it is well established that simultaneous CSs and USs are actually less efficient at driving fear learning than when CS onset precedes the US and the two co-terminate tens of seconds later. The efficacy of the latter approach, most commonly used in fear conditioning studies, is in blatant contradiction with the Hebbian rule because single-unit recording studies have revealed that the CS-evoked activity of principal LA neurons adapts quickly, such that when the US occurs, the firing rate of LA cells has almost returned to baseline. Together, these considerations indicate that widely distributed neuronal interactions support emotional learning and expression. Because biophysical modelling is unhindered by technical limitations, it can overcome the challenge of analysing complex neural interactions that extend beyond the amygdala and it make predictions about computational algorithms that enable behavioural adaptation to changing environment.

To understand the neural substrates of defensive behaviours, it is imperative we consider the complex interactions taking place between the various regions known to regulate responses to aversive situations and stimuli. Take the BNST for instance. On the basis of early lesion studies, the notion emerged that BNST and the CeA are differentially involved in the genesis of anxiety and fear, respectively.^73^ However, BNST and CeA are reciprocally connected and mounting evidence indicates that while BNST is not required for the genesis of rapid fear responses to discrete threats, it modulates their magnitude and specificity (reviewed in Gungor and Pare).^74^ Therefore, incorporating the interactions taking place between BNST and CeA will be essential to move forward in this field.

A similar case can be made for the prefrontal cortex. Previous work has established that two areas of the medial prefrontal cortex, the infralimbic and prelimbic regions, exert opposite influences on conditioned responding. However, depending on the task, say drug-seeking versus fear conditioning paradigms, the prelimbic and infralimbic regions respectively promote and inhibit appetitive or aversive responses.^75^ The multivalent influence of the medial prefrontal cortex over emotional behaviour is also evident in the pattern of inputs it receives from the basolateral complex of the amygdala (BLA). Indeed, while largely different subsets of BLA cells project to nucleus accumbens and BNST, both subsets send axon collaterals to the pre- and infralimbic regions (Lee and Pare, unpublished). These various examples underscore the fact that experiments and intuition will not suffice to unravel the dynamic interactions that underlie affective behaviours. Biophysical modelling can assist us in analysing such interactions. Indeed, a recent model^76^ suggests the involvement of amygdalar projections to the sensory thalamic reticular nucleus in emotion guided inhibitory selection, and of the amygdala/cortico/reticulo-thalamic loops in flexible attention and decision-making.

Besides allowing investigators to simultaneously consider properties at multiple levels of analysis and at distributed sites, biophysical modelling also allows one to consider hypotheses that are impossible or impractical to test experimentally. For instance, voltage- or chemo-dependent ionic conductances and be turned or on off at precise times and in specific subsets of neurons or synapses to test particular mechanistic hypotheses. In learning paradigms, modelers can identify neurons that acquire potentiated responses to conditioned stimuli and rerun the simulation after selectively ablating these cells, effectively going back in time to determine whether they are necessary for learning. This approach was used successfully in the amygdala and perirhinal cortex to show that when ‘engram’ neurons are ablated, others emerge to support memory, highlighting the fact that competitive neuronal interactions underlie learning.^31,42,68^

## Conclusions

Decades of research on the role of individual brain regions in fear learning and memory have provided pivotal findings on the amygdala as the key player in detecting threats and mediating adaptive response to negative valence. With the advent of circuit mapping and manipulation technologies, contemporary studies in animals revealed that parallel circuits within the amygdala, synaptic plasticity within these circuits as well as amygdala-projecting cortical and thalamic neurons all take part in negative valence behaviours. Although these studies paved the way for human functional brain imaging to test the hypothesis that abnormal functional connectivity is linked to maladaptive processing of aversive stimuli, we still have a superficial understanding of the key properties that enable the amygdala and associated networks to generate aversive behaviours. Consequently, our ability to link dysfunctions in these circuits to mental disorders is highly limited, resulting in ineffective therapeutic strategies.

Theoretically driven approaches and computational modelling constitute promising tools to address these difficult questions because they can identify interpretable relations between variables and quantify properties of complex systems across levels of analysis. Given the neuronal diversity and complex neurophysiological interactions within the amygdala and in its multiple targets, computational modelling is ideally suited to address questions that are beyond the reach of intuition or the experimental method.

In this review, we offer several examples of unresolved questions and opportunities in the field where computational modelling can complement experiments and quantify properties of amygdala circuits that go beyond correlative analysis of a specific measure to behaviour. For example, biophysically realistic neural circuit modelling can help determine the nature of short-term and long-term synaptic plasticity in defined cell types, and explain the functional consequences of neurotransmitter release on synaptic plasticity and fear learning. Inferences from combined computational modelling and experiments can quantify how CS and US information is represented by amygdala neurons and its targets while advancing our understanding of the neural processes involved in transforming specific stimuli into behavioural flexibility.

To address these and other challenging problems, and to relate properties of amygdala and parallels circuits in animals and humans, the field would benefit from the integration of computational modelling in both basic and translational research programs. By incorporating computational frameworks in experimental designs, we may be able to achieve a cross-level understanding of dynamics, computation and neurobiological mechanisms of fear learning as well as facilitate the development of more specific theories to explain the link between dysregulation in the broader amygdala circuits and psychiatric disorders. We are confident that this approach, ‘Computational Psychiatry,’^35^ will lead to important new insights in the near future.

## Developing a neural network model (using fear model example in Kim *et al*.^40^)

- As a first step, biophysical models are developed for all single cells (principal cell and interneuron types) using experimental data including passive properties and responses to current injections.^57^ The single-cell models include ionic channels, synapses and neuromodulators effects.^77,78^
- A suitably scaled network model is then developed. Using experimental connectivity estimates, units are connected, via excitatory or inhibitory synapses, and known synaptic delays. Estimates of both intrinsic and extrinsic connectivity have proven difficult to obtain for some of the complex distributed circuits;^58^ this makes investigations via modelling an attractive alternative to predict possibilities and complement experiments.
- Guided by the experimental literature,^32^ the excitatory and inhibitory model synapses are then endowed with short- and long-term activity-dependent plasticity and neuromodulator receptors are placed on synapses and cell bodies.
- The network model is then subjected to an experimental protocol.^79^ For variables where experimental data are scarce, the model is run iteratively to determine parameter values that match experimental unit *in vivo* data.^51,79^
